# Validation of the Personal Attributes Questionnaire-8: Gender Expression and Mental Distress in the German Population in 2006 and 2018

**DOI:** 10.3389/ijph.2022.1604510

**Published:** 2022-03-18

**Authors:** Ana Nanette Tibubos, Daniëlle Otten, Manfred E. Beutel, Elmar Brähler

**Affiliations:** Department of Psychosomatic Medicine and Psychotherapy, University Medical Center of the Johannes Gutenberg-University Mainz, Mainz, Germany

**Keywords:** sex, gender, gender expression, femininity, masculinity, mental health, epidemiology

## Abstract

**Objectives:** Mental health differences between men and women can be attributed to sex or gender. Due to absence of brief assessments, contributions of gender expressions to the mental health gap between men and women have been understudied. The purpose of this study is to develop and validate a short screening measure of gender expression and test its associations with mental distress.

**Methods:** German representative survey data from 2006 (N = 2,507) and 2018 (N = 2,516) were analysed. A short form of the Personality Attributes Questionnaire with 8 items (PAQ-8) was assessed to measure femininity and masculinity. Validity of the PAQ-8 was tested and associations between femininity, masculinity and mental health were examined.

**Results:** PAQ-8 was a valid screening measure to assess gender expression. Compared to 2006, femininity increased in women and decreased in men in 2018. Higher levels of femininity and masculinity were associated with lower distress. Sex was no longer predictive for mental distress when femininity, masculinity, age and equivalised income were considered.

**Conclusion:** Our findings support the use of gender measures, which may be more predictive of mental health than sex.

## Introduction

Mental disorders affect a large proportion of the population affecting quality of life and life expectancy [1]. Representative German studies have shown higher prevalence rates of mental disorders for women compared to men; one in three women and one in four or five men had a diagnosis of a mental disorder in the previous 12 months [2]. A variety of factors underlie this observation, e.g., genetic and biological vulnerabilities, environmental factors such as daily stressors, and differences in emotion regulation and stress responsiveness [3]. In general, mental health differences between women and men can be attributed to sex or gender differences. While sex refers to a biological construct [4], gender subsumes psychosocial variables that characterize women and men and their life contexts [5]. Sex and gender interact in the development of diseases (e.g. [6, 7]). Nonetheless, sex and gender have been used interchangeably or altogether neglected in studies on mental health [4, 8, 9].

Gender is nowadays considered as a multidimensional concept based on individual, interactional and macro areas of social life [10]. One aspect of gender are masculine and feminine traits. Based on the seminal work of Sandra Bem in 1974 [11], masculine traits such as being assertive or aggressive and feminine traits such as being affectionate and sympathetic, were assessed in a questionnaire. Initially described as a bipolar one-dimensional personality trait ranging from femininity to masculinity, gender roles were later measured as a two-dimensional concept with two separate and independent dimensions [12–14]. The dimension femininity includes scales assessing expressivity or communion capturing similar content, the term masculinity is used interchangeable with instrumentality or agency [11]. In personality psychology research, communion and agency have been referred to as the Big Two [15, 16].

Mental disorders have become a key challenge for health care in the 21st century [17] with a 1-year prevalence of over 30%. Large cross-sectional population surveys have advanced our knowledge of how common mental disorders differentially affect and burden men and women [17–19]: Compared to men, women suffer more frequently from depression, anxiety disorders, somatoform disorders, eating disorders, self-injury, suicide attempt, post-traumatic stress disorder and pill addiction, termed internalising disorders. In contrast, so-called externalising disorders are more common among men including disorders of impulse control, substance dependence, attention deficit hyperactivity disorder, overweight, alcoholism, pathological gambling, suicides and violence against others [3, 20].

Developed by Spence et al. [14], the Personal Attributes Questionnaire (PAQ) has remained one of the most frequently used assessments of gender with its main dimensions of masculinity and femininity. Masculinity and femininity can be understood as traits that reflect independent dimensions of a personality [21]. Originally, the constructs measured with this questionnaire were defined as gender roles, which can be regarded as a representation of the internalized societal gender. In the light of the shift in terminology of the gender concept [22] and in line with the definition of the American Psychological Association, we understand the Personal Attributes Questionnaire as an indicator of *gender expression* [23]. Gender expression refers to an individual’s presentation and behavior in which a person communicates its gender within a given culture. Someone’s gender expression may or may not reflect a person’s gender identity.

In previous studies, both masculinity and femininity were positively related to indicators of adjustment (low distress, self-esteem), however, higher negative correlations were found between masculinity (vs. femininity) and distress (e.g. [13, 24]). The aim of this study was twofold. The first goal was to develop and validate via item, scale, principal component, and factor analyses a brief screener for gender expression based on the PAQ which can be used in large scale surveys. Second, in order to validate the screener taking societal influences into account, the association of the dimensions masculinity and femininity with mental distress over time was assessed. As gender is both an important part of the individual self-concept and a reflection of societal trends of gender roles, we reapplied the scale in two representative national samples at two different time points, in 2006 and 2018. The shift towards a more individualistic society [25], in which values such as autonomy, self-expression and an ethos of individual rights are emphasized, expressions of diversity are more common [26, 27]. We therefore expected stronger expressions of masculinity and femininity in 2018 compared to 2006.

## Methods

### Sample

Data of two German representative studies with the same recruitment procedure conducted in the years 2006 (sample 1) and 2018 (sample 2) were analyzed. Sample 1 was split in two halves: sample 1a for short scale development, sample 1b for factorial cross-validation of the short scale. The samples were representative in terms of age, sex and educational level for the general German population, collected by an independent agency (USUMA, Berlin) in a nation-wide survey. Following a random route procedure on 258 non-overlapping sample point regions, target households were randomly selected within these areas. Subsequently, one of the household members of this address was selected by chance (Kish-Selection-Grid [28]) and contacted. The target person participated in a face-to-face interview conducted by a trained interviewer and additionally independently filled out several questionnaires. Representativeness of each sample was furthermore secured by comparisons with census data from the German Federal Statistical Office. The inclusion criteria for the study were the fluency and understanding of the German language, and a minimum age of 14 years. Of the 4,036 addresses and subjects initially selected for sample 1, 597 persons (14.8%) were not present on the three occasions when the interviewer visited the address, 899 persons (22.3%) rejected participation, and 33 people (0.8%) could not participate due to severe health issues. This led to a response rate of 62.1% with a total sample of N = 2,507 (1,357; 54.1% females) persons with a mean age of 48.0 (SD = 18.1). For sample 2 a total of 5,418 households were approached, 22.4% of these households refused to identify the person of target, and 12.7% of the target persons refused to participate. In total N = 2,516 (1,372; 54.5% females) target persons participated in the study with a mean age of 48.0 (SD = 17.6). Some respondents did not answer all items of the main construct PAQ or PAQ-8. Since there were only few missing items, mean scores for femininity and masculinity could be calculated for all respondents.

All participants received a detailed data privacy statement by the study assistant, informing about the study procedures, data collection, and anonymization of all personal data. The present study posed a low risk to the participants, as procedures such as medical treatments, invasive diagnostics or procedures causing psychological, spiritual or social harm were not included in the present study. Therefore, all participants provided verbal informed consent, which was noted by the trained interviewer before starting with the survey. In the case of minors, participants gave informed assent with informed consent being provided by their caregivers. The completed questionnaires were linked to the socio-demographic data, but did not contain any data to identify the participant. The study and procedure, including the consent procedure, were approved by the institutional ethics review board of the University of Leipzig (Az 132/18-ek). Furthermore, the study adhered to the guidelines of the ICC/ESOMAR International Code of Marketing and Social Research Practice.

### Measures

All following measures were available for both measurement points, except PAQ which was only assessed in 2006.

*PAQ and PAQ-8.* For the present study, the German version [13, 29, 30] of the PAQ (GEPAQ) with the scales masculinity/positive instrumentality and femininity/expressivity with eight items each was used in sample 1 as starting point for scale reduction. For masculinity/positive instrumentality the items of the PAQ (items of the PAQ-8 indicated in brackets) were: not at all independent—very independent, very passive—very active, not at all competitive—very competitive, can make decisions easily—has difficulty making decisions, never gives up easily—gives up very easily (PAQ-8), not at all self-confident—very self-confident (PAQ-8), feels superior—feels very inferior (PAQ-8), and goes to pieces under pressure–stands up well under pressure (PAQ-8). For femininity/expressivity the items were: not at all emotional—very emotional (PAQ-8), not at all able to devote self completely to others—able to devote self completely to others, very rough—very gentle, not at all helpful to others—very helpful to others, not at all kind—very kind, not at all aware of feelings of others—very aware of feelings of others (PAQ-8), not at all understanding of others—very understanding of others (PAQ-8), and very warm in relations with others—very cold in relations with others (PAQ-8). Each item had a six-step response answer scale ranging from 1 to 6. These extremes indicated the characteristic applying perfectly to the respondent. The closer to the middle of the scale, the more neutral the respondent answered between both opposing characteristics.

The PAQ showed a two-factor structure with moderately correlated subscales (*r* = 0.48). Reliability of the PAQ was good, with Cronbach’s α: masculinity-scale: 0.77; femininity-scale: 0.84. A brief version (PAQ-8) has been developed in this study based on the German PAQ version with 8 items in total, 4 items for masculinity and femininity each. Detailed psychometric properties and item formulation are reported in the results section (see Table 1).

**TABLE 1:** Items and descriptive statistics of the Personal Attributes Questionnaire-8 (Germany, 2006 & 2018).

GEPAQ^a^ item nr.	PAQ-8 item nr.	German	English	Scale	2006		2018	
					M^b^	SD^c^	M	SD
2	1	nicht gefühlsbetont—sehr gefühlsbetont	not at all emotional—very emotional	F	4.37	1.10	4.38	1.27
8	2	der Gefühle anderer nicht bewusst—der Gefühle anderer bewusst	not at all aware of feelings of others—very aware of feelings of others	F	4.50	1.10	4.45	1.21
10	3	gebe nie leicht auf—gebe leicht auf	never gives up easily—gives up very easily	M (inverse)	4.59	1.24	4.46	1.32
11	4	nicht selbstsicher—sehr selbstsicher	not at all self-confident—very self-confident	M	4.40	1.19	4.41	1.22
13	5	fühle mich überlegen—fühle mich unterlegen	feels superior—feels very inferior	M (inverse)	3.79	1.02	3.74	1.09
14	6	nicht verständnisvoll gegenüber anderen—verständnisvoll gegenüber anderen	not at all understanding of others—very understanding of others	F	4.52	1.10	4.69	1.14
15	7	sehr herzlich in den Beziehungen zu anderen—sehr kühl in den Beziehungen zu anderen	very warm in relations with others—very cold in relations with others	F (inverse)	4.37	1.11	4.19	1.36
16	8	kann Druck nicht standhalten—kann Druck gut standhalten	goes to pieces under pressure—stands up well under pressure	M	4.37	1.18	4.34	1.28

Note. For 2006 the first half of the sample (sample 1a, PAQ-8, development sample) was used. ^a^ GEPAQ, German version of the PAQ; ^b^M = mean scores; ^c^ SD, standard deviations.

*PHQ-4*. The Patient Health Questionnaire-4 (PHQ-4) [31] is an ultra-brief and reliable (Cronbach’s alpha = 0.80) screener for depressive symptoms and anxiety [32]. Depression items assess depressed mood and loss of interest (PHQ-2). Anxiety includes the two screening items of the Generalized Anxiety Disorder-7 [33]: “Feeling nervous, anxious or on edge” and “not being able to stop or control worrying.” The frequency of occurrence in the past 2 weeks was rated from 0 = “not at all”, 1 = “several days”, 2 = “over half the days”, and 3 = “nearly every day”. For the analyses, a single factor solution was used as a measure of distress.

*Socio-demographic variables.* Sex (male = 1, female = 2), age (stratified in groups in descriptive statistics 14-20/21-30/31-40/41-50/51-60/61-70/71+ years or as continuous variable), education (in years), and equivalised income defined as household income/√(people in household (stratified in tertiles in descriptive statistics or as continuous variable) were taken into account in analyses.

### Statistical Analyses

#### Development and Factorial Validation of PAQ-8

In order to shorten the PAQ-questionnaire, content-based item analyses to identify redundant and less comprehensible items was conducted. Four items are supposed to be eliminated for each subscale. Afterwards, item and scale analyses and principal component analysis (PCA) using varimax rotation with half of sample 1 (sample 1a) were conducted. For cross-validation, a confirmatory factor analysis (CFA) was performed with the other half of sample 1 (sample 1b). For CFA the robust maximum likelihood method with Yuan-Bentler’s scaled χ^2^ was used for model estimation [34]. Goodness of fit was evaluated by applying the following model-fit indices: the minimum discrepancy divided by its degrees of freedom (CMIN/DF), the comparative-fit-index (CFI), the Tucker-Lewis Index (TLI), standardized root mean square residual (SRMR), and the root mean square error of approximation (RMSEA) with confidence intervals (CI). For a good model fit, the ratio CMIN/DF should be as small as possible. A good model fit is shown by a non-significant YB- χ^2^ value (*p* > 0.01) as well as RMSEA ≤0.05 and CFI ≥0.97, an acceptable model fit is available if χ^2^-value/*df* ≤ 3, RMSEA ≤0.08 and CFI ≥0.95 [35]. The latent factors were used as scaling variable.

#### Construct Validation of PAQ-8

First, we compared the correlation patterns of PAQ-8 (sample 1 and 2) and PAQ (sample 1). Second, in terms of convergent and discriminant validity, correlations between PAQ-8, PAQ and distress (PHQ-4) were compared for the overall sample and stratified by sex. Third, in order to capture potential societal influences on gender expression over time, we performed multivariate analysis of variance (MANOVA) with femininity and masculinity as dependent variables (predictors: sex, year, and their interaction), and multiple regression analyses with mental distress as dependent variable for 2006 and 2018 each (predictors: sex, femininity, masculinity, and their interaction; covariates: education, equivalised income, and age). Effect sizes were reported based on partial η^2^: small <0.01, medium <. 06, large <0.14 [36], *p* <0 .05 was considered significant.

The software programs SPSS version 24 and Mplus version 8 [37] were used.

## Results

### Development and Factorial Validation of PAQ-8

**FIGURE 1:**
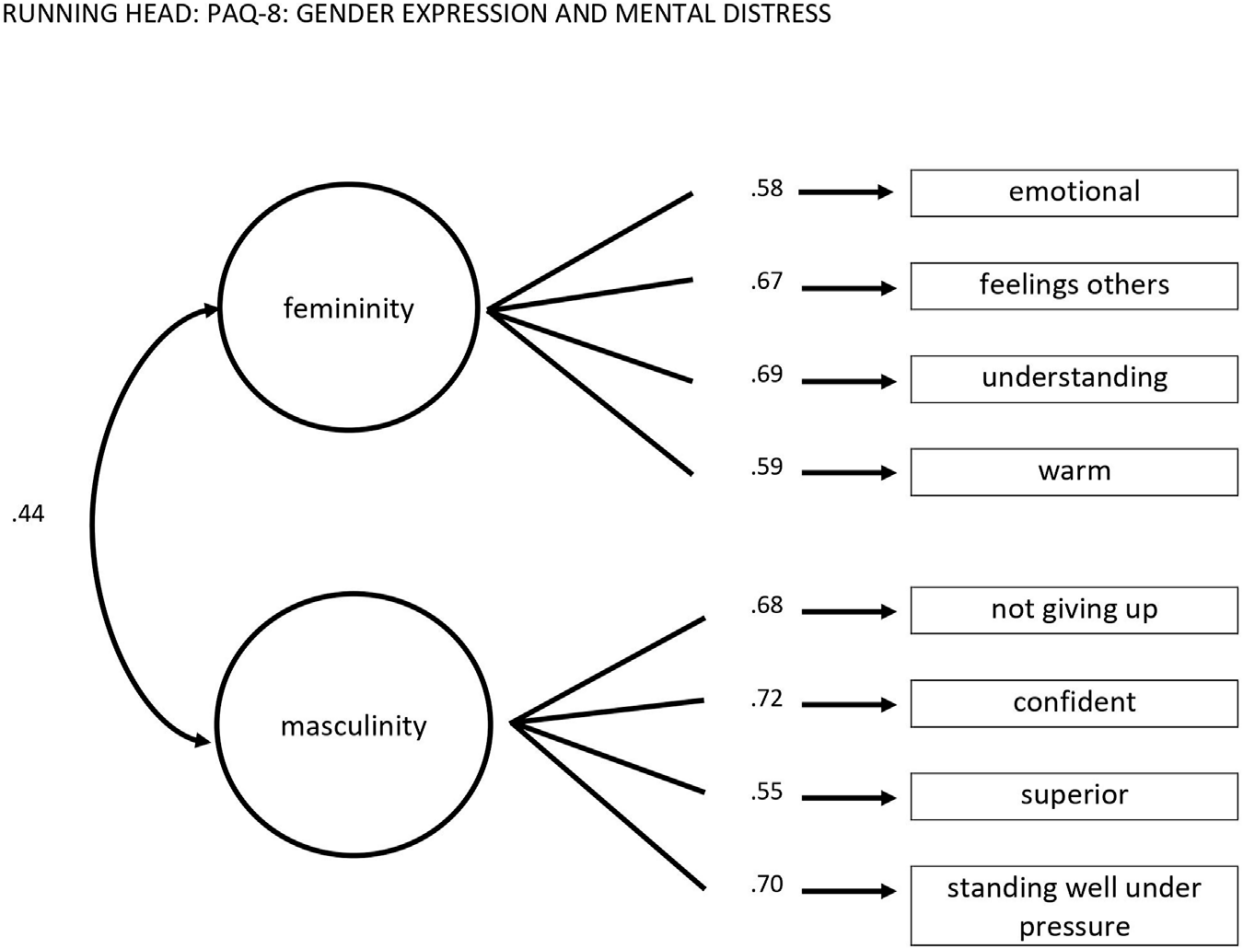
The Personal Attributes Questionnaire-8 two-factor model with standardized coefficients (Germany, 2006).

According to the objective, eight items were suggested to be excluded due to content redundancy and less comprehensible item formulations. An overview of descriptive statistics of the remaining items are displayed in Table 1. Principal component analysis pointed out two components (57.7% explained variance) based on scree-plot, Kaiser-Eigenvalues, and Minimum Average Partial test (MAP-analysis). The two theoretical dimensions femininity and masculinity were represented by the two components. Component loadings for femininity ranged from 0.71–0.72, for masculinity from 0.73 to 0.79. Also CFA results supported the two-factor model: χ^2^(19) = 90.49, *p* < 0.001, CFI = 0.97, RMSEA = 0.05 [CI 0.04 -0.06], SRMR = 0.03. The two scales (r = 0.44) with 4 items each had an acceptable internal consistency, Cronbach`s α: masculinity-scale: 0.79; femininity-scale: 0.71 and McDonald`s ω: masculinity-scale: 0.76; femininity-scale: 0.71. Figure 1 displays the results of the CFA with standardized parameters.

### Femininity and Masculinity in the German General Population Over Time

Descriptive statistics of femininity and masculinity levels for 2006 and 2018 are displayed in Supplementary Table S1. Femininity scores were higher among women, while masculinity scores were higher among men for both measurement points. Femininity scores between women and men showed larger differences in 2018 compared to 2006, with higher femininity scores for women and lower femininity scores for men. For both dimensions, comparable scores were observed between age groups. Whilst significant differences for masculinity with very small effect size were observed, no significant differences were observed for femininity in 2006. In 2018, similarly negligible effect sizes were observed for significant differences for femininity and masculinity between the age groups. In general, a non-linear trend with the highest scores for the age range of 21–40 years were revealed for both measurement points for the total sample as well as in sex-stratified analyses. Regarding education in 2006, significant differences for femininity and masculinity with small effect sizes were reported for the total sample, for women, and for men. Larger effect sizes were displayed by men. Individuals with least years of education displayed the lowest femininity and masculinity scores. In 2018, however, no significant differences were found between the educational groups for femininity. With respect to masculinity in 2018, significant differences were observed similar to 2006. Finally, analyses of gender identities for different equivalised income groups revealed slight differences between both measurement points. In 2006, gender identities of women and men did not differ between different income groups. Yet, in 2018, income groups were associated with gender expression of women. Especially masculinity scores increased along with higher income of women.

**FIGURE 2:**
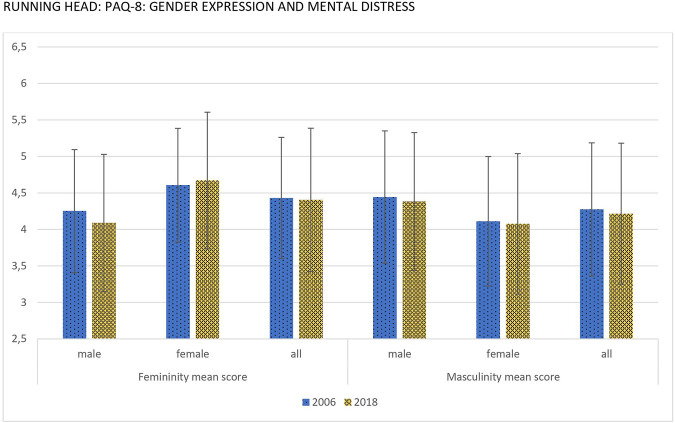
Mean scores and standard deviation of gender expressions for males, females and total sample (Germany, 2006 & 2018).

Figure 2 compares the scores of men and women in the two gender expression subscales in 2006 and 2018. A MANOVA with femininity and masculinity as dependent variables and year, sex, and the interaction as predictors was performed in order to test sex- and time associations with gender expression. The overall model did not turn out to be significant: R^2^ = 0.03, F (2,5010) = 0.187, *p* = 0.830. Femininity was consistently higher in women and masculinity higher in men in both surveys [F_Femininity_ (1,5011) = 385.76, *p* ≤ 0.001, η_p_^2^ = 0.07, F_Masculinity_ (1,5011) = 140.60, *p* ≤ 0.001, η_p_^2^ = 0.03]. As the interaction year X sex shows, in 2018 femininity was higher in women and lower in men compared to 2006 [F_Femininity_ (1,5011) = 25.37, *p* ≤ 0.001, η_p_^2^ = 0.01]. Thus, sex differences increased regarding femininity over time, but not regarding masculinity [F_Masculinity_ (1,5011) = 0.30, *p* = 0.863, η_p_^2^ = 0.00]. There was also no significant association regarding year [F_Femininity_ (1,5011) = 0.27, *p* = 0.601, η_p_^2^ = 0.00, F_m_ (1,5011) = 0.20, *p* = 0.651, η_p_^2^ = 0.00].

### Mental Distress by Sex and Gender Expressions in 2006 and 2018

In terms of convergent and discriminant validity, Tables 2, 3 present bivariate Spearman correlations between PAQ (only in 2006), PAQ-8, and PHQ-4, sex, age, education, and equivalised income. The correlation between PAQ-8 and mental distress was lower compared to the correlation between PAQ-8 and its original version. There was a stronger correlation between femininity and masculinity in the German general population in 2006 (*r* = 0.34, *p* < 0.001) compared to 2018 (*r* = 0.19, *p* < 0.001). Femininity was negatively associated to mental distress in 2006 (*r* = −0.12, *p* < 0.001) and 2018 (r = −0.14, *p* < 0.001). Masculinity was also negatively associated to distress (2006 *r* = −0.29, *p* < 0.001), but to a larger degree, particularly in 2018 (*r* = −0.39, *p* < 0.001). While the correlation between sex and masculinity was quite comparable for 2006 and 2018 (*r* = −0.17/−0.16; both *p* < 0.001), the association between femininity and sex increased (*r* = 0.22/.31, both *p* < 0.001). These significant results even hold when applied a conservatively estimated adjusted p-value of α/n (0.01/72) = 0.0001 for multiple testing since the largest p-value of the reported correlation coefficients was *p* = 0.0000000058. Comparable levels were reported referring to age and gender expression for the total sample at both measurement points. The older people were, the lesser femininity and masculinity were reported. This trend was generally higher for women compared to men. With regards to variables of socio-economic status, education showed low positive correlations with both gender identities. However, in 2018, the correlation between femininity and education approximated zero. For equivalised income at both measurement points, higher positive associations with masculinity compared to femininity were revealed in the total sample. Generally, the associations were weaker in 2018 than in 2006, with exception for women in 2006 where both dimensions of gender expression showed similar correlation coefficients with income. Subsequent sex stratified analysis revealed a counteracting pattern change over time: While the association between masculinity and equivalised income decreased nearly halfway among men, an almost twofold increase was observed for women. Furthermore, no significant association between equivalised income and femininity was given anymore for men in 2018 which also applied for the overall sample analysis.

**TABLE 2:** Spearman correlations between gender expression, mental distress, and sex. Total sample (Germany, 2006 & 2018)

	2006 (N = 2,507)								
	1.	2.	3.	4.	5.	6.	7.	8.	9.
1. PAQ^a^-8 F									
2. PAQ-8 M	0.34**								
3. PHQ^b^-4	-0.12**	-0.29**							
4. Sex	0.22**	−0.17**	0.10**						
5. PAQ-F	0.93**	0.38**	−0.13**	0.23**					
6. PAQ-M	0.35**	0.90**	−0.32**	−0.15**	0.37**				
7. Education	0.12**	0.17**	−0.08**	−0.00	0.12**	0.20**			
8. Equivalised Income	0.07**	0.13**	−0.16**	−0.08**	0.06**	0.15**	0.24**		
9. Age	−0.06**	−0.08**	0.11**	−0.01	−0.60**	−0.12**	−0.29**	−0.10**	
	2018 (N = 2,516)								
	1.	2.	3.	4.	7.	8.	9.		
1. PAQ^a^-8 F									
2. PAQ-8 M	0.19**								
3. PHQ^b^-4	−0.14**	−0.39**							
4. Sex	0.31**	−0.16**	0.07**						
5. PAQ-F	X^c^	x	x	x	x	x	x		
6. PAQ-M	x	x	x	x	x	x	x		
7. Education	0.02	0.15**	−0.07**	−0.04					
8. Equivalised Income	0.02	0.13**	−0.12**	−0.06**	0.15**				
9. Age	−0.07**	−0.06**	0.08**	0.03	−0.21**	0.24**			

Note: ^a^PAQ, personal attributes questionnaire; ^b^PHQ, patient health questionnaire, full PAQ questionnaire only available in 2006. ^c^x = not available. *Significance: *p* < 0.05**Significance: *p* < 0.01.

**TABLE 3:** Spearman correlations between gender identity, mental distress, and sex. Stratified by sex: lower diagonal for women, upper diagonal for men (Germany, 2006 & 2018).

	2006							
	1.	2.	3.	4.	5.	6	7.	8.
1. PAQ^a^-8 F	1.00	0.39**	−0.13**	0.91**	0.40**	0.11**	0.09**	−0.02
2. PAQ-8 M	0.40**	1.00	−0.29**	0.43**	0.88**	0.16**	0.15**	−0.03
3. PHQ^b^-4	−0.16**	−0.27**	1.00	−0.14**	−0.34**	−0.05	−0.12**	0.07*
4. PAQ-F	0.93**	0.43**	−0.18**	1.00	0.43**	0.12**	0.07*	−0.00
5. PAQ-M	0.38**	0.90**	−0.29**	0.40**	1.00	0.20**	0.19**	−0.07*
6. Education	0.13**	0.18**	-0.11**	0.13**	0.21**	1.00	0.27**	−0.21**
7. Equivalised Income	0.09**	0.09**	−0.19**	0.09**	0.11**	0.22**	1.00	−0.08**
8. Age	−0.09**	−0.12**	0.14**	−0.09**	−0.36**	−0.36**	−13**	1.00
	2018							
	1.	2.	3.	6.	7.	8.		
1. PAQ^a^-8 F	1.00	0.26**	−0.13**	0.03	0.01	−0.08*		
2. PAQ-8 M	0.25**	1.00	−0.38**	0.13**	0.08**	−0.01		
3. PHQ^b^-4	−0.19**	−0.40**	1.00	−0.08**	−0.11**	0.08*		
4. PAQ-F	X^c^	x	x	x	x	x		
5. PAQ-M	x	x	x	x	x	x		
6. Education	0.02	0.17**	−0.07*	1.00	0.18**	−0.14**		
7. Equivalised Income	0.06*	0.16**	−0.13**	0.13**	1.00	0.21**		
8. Age	−0.08**	−0.09**	0.08**	−0.27**	0.26**	1.00		

Note: ^a^PAQ, personal attributes questionnaire; ^b^PHQ, patient health questionnaire, full PAQ questionnaire only available in 2006. ^c^x = not available. *Significance: *p* < 0.05**Significance: *p* < 0.01.

While in univariate analyses sex was predictive of distress, which was higher in women for both measurement points, in the multivariate model with gender expression and sex as predictors, sex was not predictive of distress any more (see Table 4 and Supplementary Table S2). Femininity and masculinity explained 11% (2006) and 20% (2018) of the observed variance. Moderate negative associations of masculinity with mental distress were revealed, with slightly higher levels in 2006 compared to 2018. Also, negative associations between femininity and mental distress were observed, but with similarly low effect sizes for both measurement points. For the year 2018, there was an additional significant interaction between femininity and sex, albeit with weak effect size. The interaction pointed out a stronger negative association between femininity and mental distress among women compared to men. In sum, more observed variance was explained by the predictor set in 2018 (21%) vs. 2006 (12%). While education turned out be an irrelevant predictor in the full model, age positively and equivalised income negatively predicted mental distress with low effect sizes in 2006 and even lower for 2018.

**TABLE 4:** Multiple Regression analyses on mental distress by sex and gender expression adjusted for sociodemographic features, Model 4 (Germany, 2006 & 2018).

		Model 4			
		ß	CI^a^_LB_^b^	CI_UB_^c^	p
2006	Sex	0.050	0.010	0.090	0.015
	Gender expression				
	Femininity	−0.067**	−0.109	−0.026	0.002
	Masculinity	−0.267**	−0.309	−0.226	0.000
	Interactions				
	Femininity x Sex	−0.029	−0.071	0.013	0.178
	Masculinity x Sex	0.015	−0.027	0.057	0.479
	Sociodemographics				
	Age	0.089**	0.047	0.130	0.000
	Education	0.003	−0.022	0.028	0.833
	Equivalised income	−0.064**	−0.087	−0.041	0.000
		corr R^2^ = 0.121, ΔF = 13.762, *p* <0 .000			
2018	Sex	−0.004	−0.026	0.017	0.691
	Gender expression				
	Femininity	−0.037**	−0.059	−0.015	0.001
	Masculinity	−0.231**	−0.253	−0.210	0.000
	Interactions				
	Femininity x Sex	−0.027	−0.049	−0.005	0.014
	Masculinity x Sex	−0.002	−0.023	0.019	0.877
	Sociodemographics				
	Age	0.002**	0.001	0.004	0.000
	Education	0.007	−0.005	0.019	0.249
	Equivalised income	−0.034	−0.046	−0.023	0.000
		corr R^2^ = 0.211, ΔF = 13.270, *p* <0 .000			

Note: ^a^ CI, confidence interval, ^b^ LB, lower bound, ^c^ UB, upper bound. ** Significance: *p* < 0.01.

## Discussion

Following the recommendations of public health agencies such as the US funding guidelines of the National Institutes of Health [38], the Canadian Institute of Gender and Health, or the Robert Koch Institute in Germany [39], we provided new impetus for sex- and gender-sensitive health research. We developed an economical version of the PAQ with a total of eight items, with good internal consistency as measure of reliability for group comparisons. Its brevity makes it highly suitable for epidemiological research. The validity of the PAQ-8 is supported by our findings: As in the long version, men and women were discriminated by their measures of masculinity and femininity, respectively.

Both dimensions of gender expression showed the highest levels in the early to middle adulthood. Regarding time related aspects, the comparison between the two cohorts indicated that femininity scores were in sum higher than masculinity scores in the total sample at all measurement points. No time effect could be observed between 2006 and 2018 at the mean scores level for the German general population. An interaction between time and sex, though with very small effect size, was shown for femininity. Accordingly, sex differences increased over time with women endorsing more and men less feminine traits. An explanation for this could be a strong shift in gender terminology over the last years [22]. Furthermore, due to the inability to select a non-binary or diverse gender in this study, individuals concerned might have withdrawn from the study. Another explanation could be the discouragement to explore and display feminine traits, which is especially present among men [40]. In a meta-analysis among US college students, Donnelly and Twenge [41] observed contrary effects, specifically, a temporal increase of women’s masculinity and decrease of femininity for over 30 years. However, direct comparisons are limited due to different study populations in terms of culture, age and time range.

Furthermore, findings pointed out an increase of the gender gap. For instance, the positive association between masculinity and femininity in the German population decreased nearly by half over time for the total sample and in sex-stratified analysis. In general, masculinity was more positively associated with higher equivalised income than femininity, with the exception for women in 2006. While among men the association between masculinity and equivalised income decreased almost by half over time, the association between equivalised income and masculinity increased almost twofold for women. No association between femininity and equivalised income in men and in the overall sample was found in 2018. Contrary findings for masculinity in women and men regarding income may point to changing gender roles in society. While the stereotype of masculinity and money [42] more likely applied to men in 2006, this seemed to have shifted to women in 2018. While for women displaying more masculine traits is rewarded by society (e.g., strong association with equalized income), feminine traits are in general discouraged due to a devaluation of femininity in society, since masculinity is the norm and dominant over femininity [43], also known as femmephobia [44].

As in previous studies, both dimensions of gender expressions were negatively associated with distress [13, 24], e.g., higher levels of femininity and masculinity were associated with lower mental distress. Especially masculinity reflects resilient features showing two to three times higher negative associations than femininity to distress in bivariate and multivariate analyses. However, the association between masculinity and mental distress could be explained through the concept of toxic masculinity, which distinguishes “toxic” traditional masculine traits such as aggression and self-entitlement from “healthy” masculinity [45, 46]. Externalising disorders which are more frequently exhibited by men are associated with stigmatization of mental disorders [47]. Stigma on mental health problems in turn prevents people from reporting mental health problems [48]. Gender expression turned out to be a good predictor of distress, even when age and income were included. The fact that sex was no longer a significant predictor of mental distress when masculinity and femininity were included in the model indicates that associations between sex and mental distress are mediated by gender expression. In 2018, we found a significant interaction, yet with very small effect size, between femininity and sex. The interaction showed a trend of a stronger negative association between femininity and mental distress among women compared to men.

Based on the work of Bem [11], it has been surmised [49] that the range of behaviors available to strongly sex-typed individuals is limited (e.g., denial of weakness and need for help in men, excessive care for others while relinquishing self- interest in women). Thus, growing differentiation of cultural stereotypes between men and women might be detrimental to mental health. Further research is needed on the impact of gender dimensions such as gender expression (vs. sex) on mental health over time. In sum, findings underline that sex and gender may not be equated. We can only speculate, why the gender gap has widened over the period of 12 years. However, sensitivity to change of cultural conceptions of gender may also indicate the validity of the measure. Following the findings of Twenge et al. [25] the emphasis of “generation me” on being special and unique may lead to more polarized cultural perceptions of being masculine or feminine, while society has become more accepting of expression of diverse sexual identities.

Limitations of the study include the lack of non-binary categories and measuring only one aspect of gender, namely gender expression. Masculine and feminine traits are seen as gender stereotypes that support people in defining their self-concept, however many additional characteristics besides traits are linked to gender [50]. Information on diverse gender (including all persons whose gender does not conform to their society’s norms or values when it comes to their gendered physicality, gendered identity, gender expression or combination of those factors [51, 52]) was not present in the data and could therefore not be included in this study. Furthermore, individuals can exhibit strong masculine and feminine traits at the same time, which is indicated as androgynous [11]. Within the Sexual Configurations Theory it is argued that gender expression contains three categories, namely masculinity, femininity and androgynous gender [53] and that gender expressions exist besides sex and gender. Someone’s gender expression therefore does not necessarily match someone’s gender [53]. Androgynous, referring to an outward appearance of indeterminate gender, is not a requirement for a non-binary gender identity and women exhibiting strong masculine traits or men exhibiting strong feminine traits could identify as non-binary, women or men. Due to the shift in terminology of the gender concept, the number of persons identifying as non-binary increased over the last years [53]. In this study including data from 2006 to 2018, non-binary categories going beyond the categories of being a man or a woman were not assessed in the surveys. Additionally, we did not examine androgynous gender separately, since it did not appear as a separate scale in our principal component analyses. Furthermore, although the data sampling method was a random procedure and the response rate for both years was over 60%, refusal of participation could have led to a selection bias and should be considered when interpreting these results. Also, the cross-sectional nature of the analyses prevents one to infer causality. Strengths of the study refer to the large and representative samples from the German general population. While the samples were drawn according to the same criteria and selection process, the two assessments included somewhat different cohorts and cannot be interpreted as longitudinal data.

### Conclusion

Given differential burdens of mental disorders, sex-specific reporting of mental health research has become a scientific requirement. However, sex has been often confused with gender. Due to a lack of brief assessments, the contribution of gender to the mental health gap between men and women has been understudied. In this study, we developed and validated a short screening measure of gender expression using representative survey data sets of Germany. Our findings support the use of gender measures, which turned out to be more strongly predictive of mental health than sex.

## Abbreviations

BSRI, bem sex role inventory; CFA, confirmatory factor analysis; CFI, comparative-fit Index; CI, confidence interval; CMIN, minimum discrepancy; DF, degree of freedom; ESOMAR, european society for opinion and market research; F/F+, femininity/expressivity; GAD-7, generalized anxiety disorder 7; ICC, international chamber of commerce; MANOVA, multivariate analysis of variance; MAP-analysis, maximum a posteriori probability analysis; M/M+, masculinity/positive instrumentality; PAQ, personal attributes questionnaire; PAQ-8, brief version of PAQ with 8 items; PCA, principal component analysis; PHQ-4/PHQ-2, patient health questionnaire with 4 or 2 items, respectively; RMSEA, root mean square error of approximation; SD, standard deviation; SRMR, standardized root mean square residual; TLI, tucker-lewis index; USUMA, unabhängiger service für umfragen, methoden und analysen.
