# Supplementary material for: Validation of the Personal Attributes Questionnaire-8: Gender Expression and Mental Distress in the German Population in 2006 and 2018
Source: Int J Public Health. 2022 Mar 18;67:1604510. doi: 10.3389/ijph.2022.1604510 (PMC8971198; doi:10.3389/ijph.2022.1604510)
Supplement: Supplementary file 1 [file DataSheet1.docx]

Supplementary Table 1. Descriptive statistics of both analysis samples for femininity and masculinity (Germany, 2006 & 2018)

| **Year 2006** | **Femininity** | | | | | **Masculinity** | | | | |
| --- | --- | --- | --- | --- | --- | --- | --- | --- | --- | --- |
|  | Overall (M/SD) | w^a^ | m^b^ | p | d | Overall (M/SD) | w | m | p | d |
| Sex | 4.49/.74 | 4.59 / .782 | 4.24 / .79 | 0.000 | 0.444 | 4.07 /.76 | 4.12 / .89 | 4.42 / .84 | 0.000 | 0.351 |
| Age group (years) |  |  |  | 0.021  w: 0.059  m: 0.539 | .155  w: .191  m: .004 |  |  |  | 0.000  w: 0.002  m: 0.000 | .238  w: .255  m: .333 |
| - 14-20 | 4.44 / .76 | 4.69 / .70 | 4.18 / .73 |  |  | 4.20 / .91 | 4.21 / 89 | 4.18 / .93 |  |  |
| - 21-30 | 4.54 / .85 | 4.70 / .79 | 4.34 / .73 |  |  | 4.36 / .86 | 4.29 /.92 | 4.45 / .78 |  |  |
| - 31-40 | 4.47 / .83 | 4.61 / .82 | 4.26 / .80 |  |  | 4.38 / .89 | 4.18 / .90 | 4.66 / .80 |  |  |
| - 41-50 | 4.45 / .82 | 4.61 / .77 | 4.26 / .84 |  |  | 4.27 / .90 | 4.13 / .91 | 4.45 / .86 |  |  |
| - 51-60 | 4.36 / .79 | 4.56 / .76 | 4.16 / .77 |  |  | 4.16 / 88 | 4.04 / .88 | 4.29 / .86 |  |  |
| - 61-70 | 4.35 / .77 | 4.47 / .79 | 4.24 / .72 |  |  | 4.30 / .86 | 4.11 / .86 | 4.48 / .83 |  |  |
| - ≥ 71 | 4.41 / .77 | 4.53 / .79 | 4.24 / .71 |  |  | 4.05 / .81 | 3.90 / .84 | 4.28 / .71 |  |  |
| Education (pupils excluded) |  |  |  | 0.000  w: 0.000  m: 0.000 | .247  w: .238  m: .238 |  |  |  | 0.000  w: 0.000  m: 0.000 | .333  w: .364  m: .320 |
| ≤ 8 years | 4.32 / .80 | 4.48 / .78 | 4.15 / .79 |  |  | 4.12 / .88 | 3.94 / .89 | 4.30 / .83 |  |  |
| 9 – 11 years | 4.53 / .78 | 4.66 / .77 | 4.34 / .74 |  |  | 4.33 / .88 | 4.22 / .89 | 4.49 / .84 |  |  |
| ≥ 12 years | 4.50 / .85 | 4.68 / .82 | 4.34 / .84 |  |  | 4.51 / .80 | 4.35 / .81 | 4.65 / .76 |  |  |
| Equivalised income |  |  |  | .538  w: 0.694  m: 0.837 | .003  w: .000 m: .000 |  |  |  | 0.048  w: 0.394  m: 0.329 | .110  w: .041  m: .090 |
| 1. Tertile | 4.46 / .82 | 4.61 / .77 | 4.25 / .85 |  |  | 4.21 / .89 | 4.09 / .90 | 4.38 / .87 |  |  |
| 1. Tertile | 4.43 / .79 | 4.61 / .78 | 4.23 / .75 |  |  | 4.25 / .87 | 4.11 / .90 | 4.41 / .81 |  |  |
| 1. Tertile | 4.42 / .79 | 4.57 / .79 | 4.26 / .78 |  |  | 4.31 / .89 | 4.17 / .88 | 4.47 / .86 |  |  |
| **Year 2018** | **Femininity** | | | | | **Masculinity** | | | | |
|  | Overall (M/SD) | w | m | p | d | Overall (M/SD) | w | m | P | d |
| Sex | 4.405/.981 | 4.694 / .883 | 4.107 / .912 | 0.000 | 0.655 | 4.215/.967 | 4.109 / .932 | 4.404 / .891 | 0.000 | 0.323 |
| Age group (years) |  |  |  | 0.002  w: 0.088  m: 0.012 | .180  w: .180  m: .238 |  |  |  | 0.012  w: 0.002  m: 0.537 | .168  w: .247  m: .003 |
| - 14-20 | 4.45 / .96 | 4.83 / .85 | 4.14 / .94 |  |  | 4.19 / 1.03 | 4.14 / 1.07 | 4.23 / .1.01 |  |  |
| - 21-30 | 4.48 / .97 | 4.78 / .90 | 4.15 / .94 |  |  | 4.29 / .91 | 4.11 / .94 | 4.48 / .85 |  |  |
| - 31-40 | 4.54 /.91 | 4.79 / .84 | 4.21 / .90 |  |  | 4.31 / .91 | 4.21 / .90 | 4.45 / .91 |  |  |
| - 41-50 | 4.51 / .93 | 4.70 / .95 | 4.26 / .84 |  |  | 4.28 / .95 | 4.18 / .95 | 4.41 / .93 |  |  |
| - 51-60 | 4.33 / .96 | 4.62 / .87 | 3.99 / .94 |  |  | 4.28 / .94 | 4.18 / .93 | 4.40 / .93 |  |  |
| - 61-70 | 4.37 / .90 | 4.60 / .87 | 4.07 / .85 |  |  | 4.19 / .88 | 4.01 / .87 | 4.39 / .85 |  |  |
| - ≥ 71 | 4.33 / .98 | 4.63 / .87 | 3.91 / .97 |  |  | 4.24 / .93 | 3.84 / .91 | 4.37 / .76 |  |  |
| Education (pupils excluded) |  |  |  | 0.058  w: 0.263  m: 0.276 | .090  w: .090  m: .090 |  |  |  | 0.000  w: 0.000  m: 0.000 | .333  w: .381  m: .278 |
| ≤ 8 years | 4.38 / .97 | 4.64 / .93 | 4.05 / .91 |  |  | 4.06 / .96 | 3.88 / .97 | 4.27 / .91 |  |  |
| 9 – 11 years | 4.48 / .95 | 4.73 / .87 | 4.15 / .94 |  |  | 4.31 / .89 | 4.22 / .87 | 4.44 / .89 |  |  |
| ≥ 12 years | 4.43 / .89 | 4.71 / .82 | 4.14 / .86 |  |  | 4.44 / .87 | 4.30 / .91 | 4.59 / .81 |  |  |
| Equivalised income |  |  |  | 0.017  w: 0.009  m: 0.167 | .110  w: .168  m: .110 |  |  |  | 0.000  w: 0.000  m: 0.048 | .238  w: .286  m: .142 |
| 1. Tertile | 4.35 /.1.02 | 4.59 / .97 | 4.04 / .1.00 |  |  | 4.09 / .98 | 3.93 / .99 | 4.30 /.93 |  |  |
| 1. Tertile | 4.48 / .88 | 4.76 / .82 | 4.16 / .85 |  |  | 4.28 / .89 | 4.14 / .90 | 4.43 / .85 |  |  |
| 1. Tertile | 4.41 / .93 | 4.73 / .86 | 4.09 / .90 |  |  | 4.36 / .90 | 4.26 / .90 | 4.46 / .88 |  |  |

Note: ^a^w = women, ^b^m = men. N_2006_ = 2,504_F_/ 2,505 _M_, N_2018_ = 2,512 _F_/2,511_M_. Inference tests used were χ2-tests (sex) and ANOVAs (age group, education and equivalised income). Effect size presented as Cohen’s d.

Supplementary Table 2. Multiple Regression analyses on mental distress by sex and gender expression adjusted for sociodemographic features, Models 1-3 (Germany, 2006 & 2018)

|  |  | Model 1 | | | | Model 2 | | | | Model 3 | | | |
| --- | --- | --- | --- | --- | --- | --- | --- | --- | --- | --- | --- | --- | --- |
|  |  | ß | CI^a^_LB_^b^ | CI_UB_^c^ | p | ß | CI_LB_ | CI_UB_ | p | ß | CI_LB_ | CI_UB_ | p |
|  |  |  |  |  |  |  |  |  |  |  |  |  |  |
| 2006 | Sex | .091** | .051 | .131 | 0.000 | .056** | .015 | .096 | 0.007 | .054** | .014 | .095 | 0.009 |
|  | *Gender roles* |  |  |  |  |  |  |  |  |  |  |  |  |
|  | Femininity |  |  |  |  | -.069** | -.111 | -.027 | 0.001 | -.069** | -.111 | -.027 | 0.001 |
|  | Masculinity |  |  |  |  | -.280** | -.322 | -.239 | 0.000 | -.281** | -.323 | -.239 | 0.000 |
|  | *Interactions* |  |  |  |  |  |  |  |  |  |  |  |  |
|  | Femininity x Sex |  |  |  |  |  |  |  |  | -.029 | -.071 | .013 | 0.178 |
|  | Masculinity x Sex |  |  |  |  |  |  |  |  | .013 | -.029 | .055 | 0.534 |
|  |  | corr R^2^=.008, ΔF=19.696, p=0.000 | | | | corr R^2^=.106, ΔF=126.590, p=0.000 | | | | corr R^2^=.106, ΔF=0.315, p=0.401 | | | |
|  |  |  |  |  |  |  |  |  |  |  |  |  |  |
| 2018 | Sex | .028 | .006 | .051 | 0.014 | .002 | -.020 | .024 | 0.879 | .001 | -.021 | .022 | 0.960 |
|  | *Gender roles* |  |  |  |  |  |  |  |  |  |  |  |  |
|  | Femininity |  |  |  |  | -.041** | -.063 | -.019 | 0.000 | -.042** | -.064 | -.020 | 0.000 |
|  | Masculinity |  |  |  |  | -.241** | -.263 | -.220 | 0.000 | -.241** | -.262 | -.220 | 0.000 |
|  | *Interactions* |  |  |  |  |  |  |  |  |  |  |  |  |
|  | Femininity x Sex |  |  |  |  |  |  |  |  | -.028** | -.050 | -.006 | 0.011 |
|  | Masculinity x Sex |  |  |  |  |  |  |  |  | -.005 | -.026 | .016 | 0.635 |
|  |  | corr R^2^=.002, ΔF=6.026, p=0.014 | | | | corr R^2^=.197, ΔF=288.959, p=0.000 | | | | corr R^2^=.199, ΔF=3.741, p=0.024 | | | |

Note: ^a^ CI = confidence interval, ^b^ LB = lower bound, ^c^ UB = upper bound. ** Significance: p < 0.01
